# MHC allele frequency distributions under parasite-driven selection: A simulation model

**DOI:** 10.1186/1471-2148-10-332

**Published:** 2010-10-27

**Authors:** Maciej Jan Ejsmond, Wiesław Babik, Jacek Radwan

**Affiliations:** 1 Institute of Environmental Sciences, Jagiellonian University, ul. Gronostajowa 7, 30-387 Kraków, Poland; 2 Institute of Systematics and Evolution of Animals, Polish Academy of Sciences, ul. Sławkowska 17, 31-016 Kraków, Poland; 3 Institute of Nature Conservation, Polish Academy of Sciences, al. A. Mickiewicza 33, 31-120 Kraków, Poland

## Abstract

**Background:**

The extreme polymorphism that is observed in major histocompatibility complex (MHC) genes, which code for proteins involved in recognition of non-self oligopeptides, is thought to result from a pressure exerted by parasites because parasite antigens are more likely to be recognized by MHC heterozygotes (heterozygote advantage) and/or by rare MHC alleles (negative frequency-dependent selection). The Ewens-Watterson test (EW) is often used to detect selection acting on MHC genes over the recent history of a population. EW is based on the expectation that allele frequencies under balancing selection should be more even than under neutrality. We used computer simulations to investigate whether this expectation holds for selection exerted by parasites on host MHC genes under conditions of heterozygote advantage and negative frequency-dependent selection acting either simultaneously or separately.

**Results:**

In agreement with simple models of symmetrical overdominance, we found that heterozygote advantage acting alone in populations does, indeed, result in more even allele frequency distributions than expected under neutrality, and this is easily detectable by EW. However, under negative frequency-dependent selection, or under the joint action of negative frequency-dependent selection and heterozygote advantage, distributions of allele frequencies were less predictable: the majority of distributions were indistinguishable from neutral expectations, while the remaining runs resulted in either more even or more skewed distributions than under neutrality.

**Conclusions:**

Our results indicate that, as long as negative frequency-dependent selection is an important force maintaining MHC variation, the EW test has limited utility in detecting selection acting on these genes.

## Background

Major histocompatibility complex (MHC) genes are the most polymorphic genes found in vertebrates, having hundreds of alleles in human populations and, generally, dozens of alleles in other vertebrate species (reviewed in [1-4]). Such extreme polymorphism is thought to be associated with the function of MHC proteins, which present antigens derived from parasites to lymphocytes, thus inciting the adaptive immune response [5]. Parasites are under evolutionary pressure to evade detection by the host immune system and are, therefore, expected to adapt most quickly to those MHC alleles that are found at the highest frequency in the host population. The negative frequency-dependent selection resulting from this situation gives a selective advantage to rare MHC alleles and can cause high levels of polymorphism to be maintained in host MHC genes [6-8]. Another mechanism that may result in maintenance of polymorphism in MHC genes is the heterozygote advantage resulting from an increased range of antigen types that may be presented by MHC heterozygotes compared to homozygotes [6,9]. Although simulations have shown that the capacity to maintain MHC polymorphism by heterozygote advantage is lower than for negative frequency-dependent selection [8,10], heterozygote advantage has received convincing empirical support from controlled multiple infection studies [11,12].

Widespread polymorphisms in MHC genes and ample evidence (reviewed in [1-4,13]) that MHC sequences in most species have evolved under positive selection (i.e., selection favoring new and, therefore, initially rare allelic variants) suggest that the selective pressures maintaining polymorphisms must be pervasive. However, detection of such pressures over short timescales often remains elusive [1,2,4,13]. One test that is routinely used to detect selection acting on MHC genes in the recent history of a population (over dozens to hundreds of generations) is the Ewens-Watterson test (EW) [2,14]. The test is based on comparing the distribution of allele frequencies in a given population to the distribution expected under neutrality. Under neutrality (i.e., when only mutation and drift are operating), most alleles will occur at low frequency, with only a few alleles drifting to relatively high frequencies [15]. Balancing selection resulting from heterozygote advantage can be expected to produce allele frequencies that are more evenly distributed than would be expected under neutrality [16], whereas directional selection should produce an allele distribution that is skewed in favor of beneficial alleles [14,17]. It is not clear, though, how allele frequencies should behave under negative frequency-dependent selection. In a recent review, Spurgin and Richardson [13] tabulated rare-allele advantage as one of mechanisms which should result in more even MHC allele distributions, but in another place they stress dynamic nature of frequency-dependent process which could explain spatio-temporal variation in heterozygosity observed in field studies (e.g. [18]). Because negative frequency-dependent selection can function to maintain polymorphisms within a population, it is often considered a form of balancing selection, However, rather than maintaining stable allele frequencies, negative frequency-dependent selection may cause cyclic selection, driving rare alleles to relatively high frequencies before they are selected against [6,7]. We hypothesized that the allele distribution resulting from such a process might often be indistinguishable from the distribution that would be expected under neutrality, or perhaps even more skewed.

The aim of our study was to compare frequency distributions of MHC alleles in simulations that mimicked host-parasite coevolution under three scenarios: 1) simultaneous action of negative frequency-dependent selection and heterozygote advantage; 2) heterozygote advantage without negative frequency-dependent selection; and 3) negative frequency-dependent selection without heterozygote advantage. We then examined how often the resulting allele frequencies significantly departed from neutrality under each scenario using EW.

## Results & Discussion

**Figure 1:**
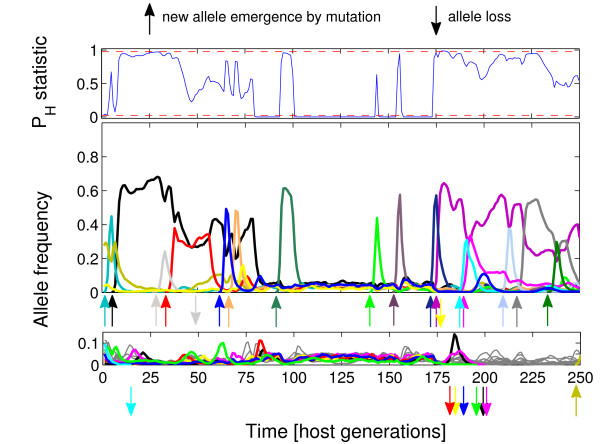
**Exemplary trajectories of P**_**H **_**statistic (top panel) and allele frequencies (middle and bottom panels) for host populations attacked by 25 pathogen species for simultaneous action of negative frequency-dependent selection and heterozygote advantage**. The blue line in the top panel represents trajectory of P_H _statistic while the red dashed lines demarcate thresholds of P_H _= 0.025 and 0.975, above (or below) which allele frequencies are considered to significantly differ from neutrality. Allele trajectories, represented in the middle and bottom panels, were split into two groups: low frequency (< 0.15) (bottom panel) and high frequency (> 0.15) (middle panel) for clarity of presentation (note that the colours of trajectories in two frequency groups may overlap). Thick colour lines represent trajectories of alleles emerging or lost from a host population during the sampled period, all other ones are in grey.

**Figure 2:**
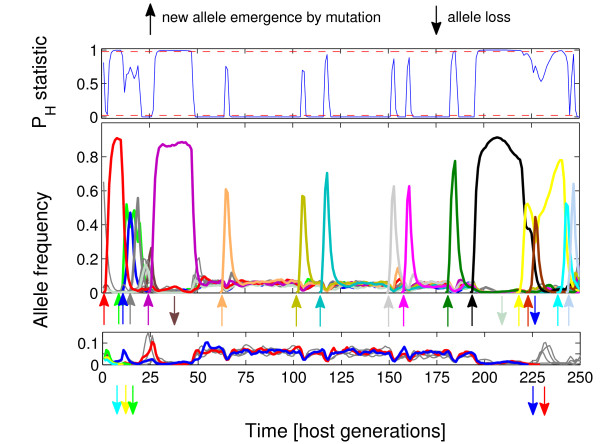
**Exemplary trajectories of P**_**H **_**statistic (top panel) and allele frequencies (middle and bottom panels) for host populations attacked by 25 pathogen species for negative frequency-dependent selection only**. The blue line in the top panel represents trajectory of P_H _statistic while the red dashed lines demarcate thresholds of P_H _= 0.025 and 0.975, above (or below) which allele frequencies are considered to significantly differ from neutrality. Allele trajectories, represented in the middle and bottom panels, were split into two groups: low frequency (< 0.15) (bottom panel) and high frequency (> 0.15) (middle panel) for clarity of presentation (note that the colours of trajectories in two frequency groups may overlap). Thick colour lines represent trajectories of alleles emerging or lost from a host population during the sampled period, all other ones are in grey.

**Figure 3:**
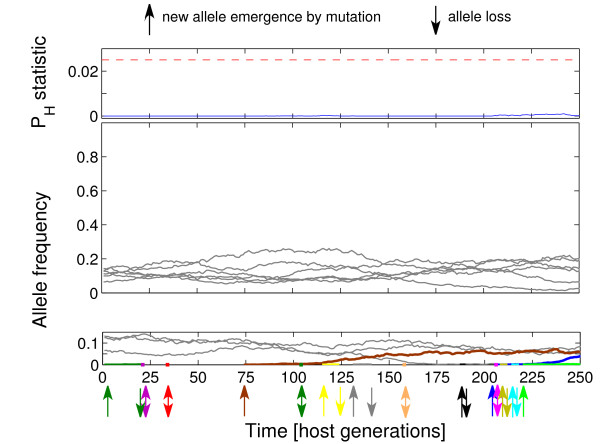
**Exemplary trajectories of P**_**H **_**statistic (top panel) and allele frequencies (middle and bottom panels) for host populations attacked by 25 pathogen species and for heterozygote advantage only**. The blue line in the top panel represents trajectory of P_H _statistic while the red dashed line demarcates threshold of P_H _= 0.025 below which allele frequencies are considered to significantly differ from neutrality. Allele trajectories, represented in the middle and bottom panels, were split into two groups: low frequency (< 0.15) (bottom panel) and high frequency (> 0.15) (middle panel) for clarity of presentation. Thick colour lines represent trajectories of alleles emerging or lost from a host population during the sampled period, all other ones are in grey.

The changes in allele frequencies under the three scenarios analysed are presented in Figure 1, 2 and 3. Under simultaneous action of negative frequency-dependent selection and heterozygote advantage (Figure 1), the alleles increased in frequency just after emergence through a mutation and might stay in relatively high frequencies, up to ca. 0.65, through a few dozens of generations (e.g. "black" allele, generations 1-100). There were also periods with very even distribution of allele frequencies (e.g. generations 100-150). After an increase in frequency of new alleles, the less frequent ones were sometimes lost (generations 180-200). Similarly, under negative frequency-dependent selection only (Figure 2), alleles increased in frequency soon after emergence, but the peak frequency could be much higher than in Figure 1, up to ca. 0.9. However, probably because of the lack of heterozygote advantage, alleles were less likely to maintain high frequency for long (note the difference in the number of sharp 'peaks' between scenarios in Figure 1 and 2). Similarly as in Figure 1, there were long periods with very even distribution of allele frequencies (e.g. generations 125-150). Under the sole action of heterozygote advantage (Figure 3), the increase in frequency of a new allele was very slow in comparison with the other scenarios. New alleles were often lost soon after emergence.

**Figure 4:**
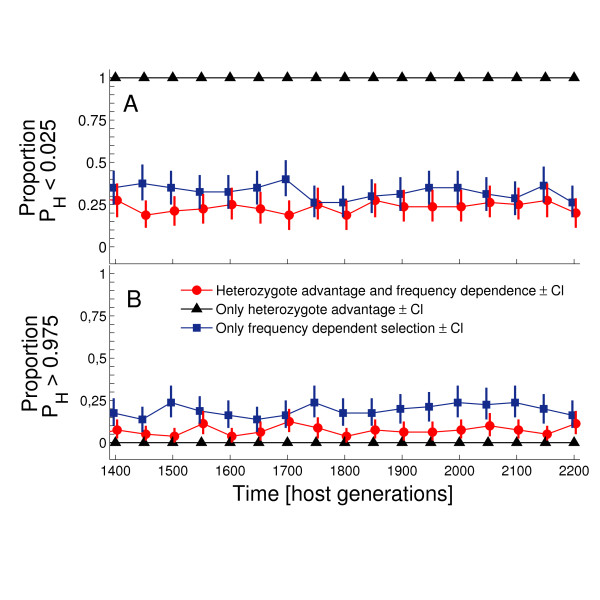
**The proportion of P**_**H **_**statistics indicating more even (A) and more skewed (B) distributions than expected under neutrality**. Populations of hosts coevolves with 25 pathogen species.

**Figure 5:**
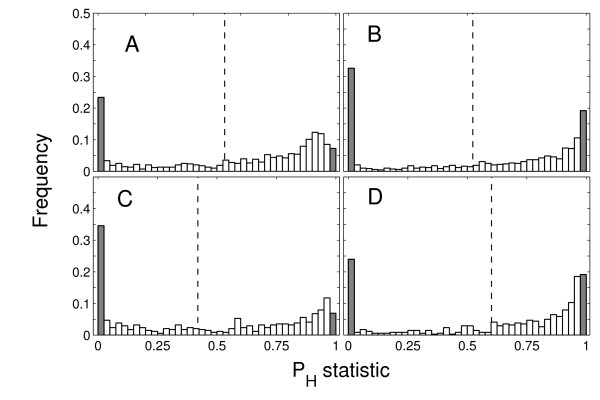
**The distributions of P**_**H **_**statistic for two scenarios: negative frequency-dependent selection and heterozygote advantage acting simultaneously (A, C), and negative frequency-dependence only (B, D)**. Shaded bars indicate the proportion of cases where P_H _< 0.025 (i.e. allele distributions more even than expected under neutrality) and P_H _> 0.975 (allele distributions more skewed than expected under neutrality). Populations of host coevolves either with 25 (A, B), or 50 (C, D) pathogen species. The samples where taken from each simulated population every 50 generations through 800 host generations, replicated 80 (A, C) or 40 (B, D) times.

The proportion of tests indicating allele distributions that were either more even or more skewed than expected under neutrality is shown in Figure 4 and 5. When negative frequency-dependent selection resulting from host-parasite coevolution was disabled, all runs resulted in frequency distributions that were significantly more even (as determined by EW) than expected under neutrality (Figure 4A). This result shows that heterozygote advantage resulting from the increased probability of an MHC heterozygote detecting a parasite assault results in allele distributions that conform to expectations from previous, more simplified models of symmetric overdominance acting on MHC [2,16]. However, when negative frequency-dependent selection was allowed to act alone, the proportion of tests that indicated a more even distribution than expected under neutrality was much lower, at approximately 33% (Figure 5B). Furthermore, a substantial proportion of tests (19%) had distributions significantly more skewed than expected under neutrality (Figure 5B). Overall, our results indicate that the effect of negative frequency-dependent selection on allele distributions is unpredictable and often indistinguishable from the effects of drift (as shown by the large proportion of non-significant tests). As shown in Figure 5, doubling the number of pathogens species coevolving with hosts did not influence qualitatively P_H _statistic distributions (see Figure 5A vs. 5C and 5B vs. 5D).

When heterozygote advantage and negative frequency-dependent selection were allowed to act simultaneously, the proportion of tests having a significant departure from neutrality was similar to that under negative frequency-dependent selection acting alone (Figure 4A and 4B). This result indicates that the dynamics of the allele distributions that were produced were dominated by negative frequency-dependent selection. Our results are consistent with results of the simulation model created by Borghans et al. [8], who concluded that negative frequency-dependent selection is a more powerful mechanism determining the maintenance of polymorphism in MHC genes than is heterozygote advantage.

The discrimination between mechanisms maintaining MHC polymorphism has long been debated (reviewed in [13,19,20]). Takahata and Nei [6] concluded that both heterozygote advantage and negative frequency dependence (which they referred to as minority advantage) can explain common features of MHC diversity such as trans-species polymorphism, high number of alleles and high heterozygosity. Higher parasite resistance of heterozygotes is often interpreted in favour of the heterozygote advantage mechanism, but as rare alleles are almost exclusively found in heterozygotes, negative frequency-dependence would result in the same pattern [12,13]. Our results indicate, however, that the two processes differ markedly in the distribution of MHC allele frequencies they produce. Indeed, it seems quite possible that the relatively low proportion of populations of various species in which allele frequencies have been found to be significantly more even than expected under neutrality (see Table two in [2]) can be explained by the common occurrence of negative frequency-dependent selection acting in these populations. However, inferring type of selection based on distributions of allelic frequencies in particular case studies would not be possible due to unpredictable dynamics of the frequency-dependent selection: in appreciable proportion of cases allelic frequencies will be more even than under neutrality, making them indistinguishable from those under heterozygote advantage.

The growing empirical evidence for associations between particular alleles and either resistance or susceptibility to infection has been suggested to be consistent with negative frequency-dependent selection driving the maintenance of polymorphism in MHC genes [4]. In reed warblers, the allele shown to be associated with the prevalence of a species of *Plasmodium *had earlier been observed to cycle between low and high frequency [21,22], which might be indicative of negative frequency-dependent selection as long as negative frequency-dependent selection is an important force maintaining MHC variation, the EW test may have limited utility in detecting selection acting on these genes (see [2] for further discussion of limitations of EW test due to other reasons). The number of parasite species simulated did not substantially affect our conclusions (Figure 5).

Results of the simulations presented in Figure 1 and 2 provide an insight into the reason why a fraction of samples showed allele distributions significantly more even, while other samples were more skewed or indistinguishable from neutrality. This inconsistency apparently resulted from dynamics of frequency-dependent selection, characterised by rapid increases in frequencies of some alleles followed by equally rapid decreases. The pattern was less dynamic under both types of balancing selection acting together (Figure 1) than under frequency-dependence acting alone (Figure 2): the periods of relatively high frequency, for a given allele, are longer and consequently the periods of even allele distributions are relatively less frequent (Figure 1). Under both scenarios the dynamics is dominated by newly emerged alleles (up-pointed arrows in Figure 1, 2 and 3) which seem to escape recognition by pathogens and to increase in frequency until pathogens "catch up" with them, which may take many generations. Changes in frequencies of comparable magnitude for alleles which were maintained in populations for a longer time were also observed (not shown on Figure 1, 2 and 3), but were relatively rare. However, apart from mutation (including microrecombination), immigration from other populations may be an important source of alleles which give resistance to local parasites. Thus, dynamics depicted in Figure 1, with rare alleles rising to high frequencies may be typical of many natural populations. Indeed, strong temporal fluctuations in MHC allele frequencies were reported in warblers [21] and soay sheep [23].

Under heterozygote advantage as a sole mechanism of balancing selection, sharp increases of allele frequencies were never observed, and newly-emerged alleles were often lost (Figure 3). This indicates that frequency-dependence and heterozygote advantage lead to high MHC polymorphism in different ways: frequency dependence is more efficient in maintaining newly-emerged alleles, whereas heterozygote advantage maintains standing variation more easily. Our simulations also suggest that strong molecular signatures of positive selection acting on MHC in many species [2,4], which arise by fixation of non-synonymous substitutions, are more likely due to frequency-dependent selection than to heterozygote advantage.

Due to high computational demands of our simulations, we used here a limited set of parameter values, carefully chosen on the basis of earlier simulations [8,24] which explored larger parameter space. Parameters used in this paper were chosen to be both biologically realistic and resulting in the level of MHC polymorphism observed in natural populations, as well as in reasonably strong negative frequency-dependent selection. For these reasons, we have not simulated high mutation rates, which lead to high levels of MHC polymorphism [8], but very weak selection on host genotypes (See Figure 1 and 3 in [24]). While results might differ under another set of parameter values (e.g. in bottlenecked populations, see [24]), we believe our result approximate mechanisms operating in large host populations interacting with a number of parasite species, and thus will apply to many populations. Importantly, our model recovered classical result that heterozygote advantage results in more even allele distributions than expected under neutrality, which gives us confidence that our choice of parameters is indeed realistic. We believe that our results concerning somewhat unpredictable allele distributions under frequency-dependence will also be quite universal, although further theoretical work is needed to explore generality of this finding.

## Conclusions

Distributions of MHC allele frequencies differ between types of balancing selections: the distribution is more even than neutral expectation under heterozygote advantage, but unpredictable, and often indistinguishable from neutrality, under negative frequency-dependent selection. Therefore neutrality tests based on the departures of allele frequency distribution from neutral expectations, such as the Ewens-Watterson test, may not be well suited to infer balancing selection on MHC genes.

## Methods

### Deriving host populations

The simulations that we ran were based on the model proposed by Borghans et al. [8] as adapted by Ejsmond and Radwan [24]. The conditions of our model were as follows: diploid hosts having a constant population size N = 3000, coevolving with populations of 25 or 50 haploid pathogen species. The two MHC molecules being carried by each diploid host were represented as 16-bit binary strings. Each bit can be thought of as representing an amino acid that is crucial for binding the antigens that subsequently get presented to T-cell receptors, for example, those that are involved in forming pockets implicated in binding specificity [25]. Each haploid pathogen was assumed to be able to produce 20 antigens, each 16-bits long. When at least seven bits of the antigen sequences matched an MHC molecule, the pathogen was presented. Consequently, a random MHC molecule had a probability equal to 0.043 of presenting one randomly generated antigen, a value close to empirical estimates [26]. The number of antigens of the same pathogen that were simultaneously presented was assumed to have no effect on the strength of the immune response produced or on host fitness.

Hosts in the simulation interacted with 25 or 50 species of haploid pathogens for 10 pathogen generations in each host generation. All pathogen species in both variants of our simulations (25 or 50 species) had population sizes equal to the size of the host populations. Each new generation of pathogens was created with haplotype frequencies proportional to the fitness gained by each haplotype in the parental generation. The fitness of a particular pathogen haplotype was proportional to the number of hosts it infected. During each generation, each host was exposed to one parasite of each parasite species. Parasite haplotypes attacking individual hosts were drawn at random in each pathogen generation with a probability equal to their proportion in the population. The fitness of the hosts was calculated as the proportion of pathogens that they recognized in a given generation. Consequently, hosts which recognized the same number of pathogens from different species had identical fitness.

After every 10 pathogen generations, a new generation of hosts was recruited by generating descendants from the host population based on host fitness, such that each descendant inherited one MHC haplotype from each of two different individuals who were drawn at random from the parental generation with the probability of receiving a haplotype from a parental host being proportional to that host's fitness. As in [8], mutations in host MHC molecules were simulated by generating new bit strings to account for the fact that variation in MHC genes often arises by micro-recombination rather than by point mutations. As in our earlier work [24], we assumed a rate of 10^-5 ^mutations per allele per host generation.

Haploid pathogens reproduced asexually, resulting in offspring and parents having identical genotypes except for where mutations occurred. Mutations in our model were simulated stochastically, so that each bit of the antigen could change to the reverse in each generation. Based on the work of Borghans et al. [8], we assumed that the mutation rates for parasites are generally higher than they are for hosts, which allow for a higher rate of evolution in parasites. These assumptions about mutational processes are somewhat oversimplified, though this appears not to influence general features of the derived populations [8]. Populations were derived with a mutation rate of 2 × 10^-3 ^per antigen per pathogen generation. This rate results in parasites exerting significant selection pressure on their hosts (at lower rates, parasites do not evolve quickly enough to adapt to hosts, whereas at higher rates, random rather than host-adapted parasite genotypes appear) and maintains realistic levels of polymorphism in simulated populations [24].

Coevolution was initiated concurrent with random generation of a population of hosts and pathogens, such that host polymorphism was maximal at the outset and then evolved to a level determined by natural selection. This method shortened the time necessary to derive host populations and produced the same results as if evolution was initiated from just two host alleles [24]. Hosts and parasites were allowed to co-evolve for 2,200 host generations, i.e., 22,000 pathogen generations.

### Scenarios of MHC evolution

In our model, we compared three scenarios: 1) simultaneous action of negative frequency-dependent selection and heterozygote advantage; 2) heterozygote advantage without negative frequency-dependent selection; and 3) negative frequency-dependent selection without heterozygote advantage.

To simulate heterozygote advantage, we set the fitness of a heterozygote carrying a pathogen recognition allele to be equal to that of a homozygote bearing the same allele [8,24]. The heterozygote was, thus, twice as likely to respond to a random parasite. Heterozygote advantage was disabled by setting the fitness of a heterozygous individual carrying a pathogen recognition allele to be equal to half of that of a homozygote bearing the same allele. Excluding negative frequency-dependent selection from the model was achieved by choosing the parasites to be recruited to the next generation at random, so that they did not have the ability to adapt to the most common host genotypes [8].

We derived 80 independent host populations for scenarios where 25 pathogen species interacted with hosts, and 40 independent replications (a number of runs was limited by computation time) for scenarios where the number of pathogen species was set to 50. Allele distributions were calculated every 50 generations in each simulation run, starting from generation 1,400, which was after the number of alleles maintained in a population had stabilized for all three scenarios, and ending at generation 2200. Based on a few runs which extended to 5000 generations (not shown) we concluded that the slot of 800 generations was enough to fully characterize dynamics of allele frequency changes (see exemplary runs in Figure 1, 2 and 3), and extending simulations would not give us more information but would limit the number of populations we sampled. All of our simulations were performed in MATLAB 7.9 (MathWorks).

### Testing for departures from neutrality

The departure of the resulting MHC allele frequencies from neutrality for each sampled generation was tested by EW, which compares observed distributions to those expected under neutrality. The test was calculated using the program developed by Slatkin [27,28]. The program generates 10^5 ^neutral sample configurations with the Stewart's [29] algorithm through Monte Carlo simulations. For each host generation sampled, the proportion of significant EW tests was recorded as well as the direction of the departure from neutrality, which could be either more even (P_H _< 0.025) or more skewed (P_H _> 0.975) than expected under neutrality. Confidence limits for P_H _presented in Figure 4 were bootstrapped from 80 replicates of scenario with 25 pathogen species.

## Authors' contributions

MJE participated in study design, performed computer simulations and helped to draft the manuscript. WB helped in design of computer simulations and helped to draft the manuscript, JR conceived the study, participated in its design and coordination and drafted the manuscript. All authors read and approved the final manuscript.
